# The emerging role of interleukin‐37 in cardiovascular diseases

**DOI:** 10.1002/iid3.159

**Published:** 2017-05-26

**Authors:** Xinyu Zhuang, Bangwei Wu, Jian Li, Haiming Shi, Bo Jin, Xinping Luo

**Affiliations:** ^1^ Department of Cardiology Huashan Hospital Fudan University Shanghai China

**Keywords:** cardiovascular diseases, inflammatory immunity response, IL‐37

## Abstract

**Introduction:**

Interleukin (IL)‐37 is a newly identified member of the IL‐1 family, and shows a growing role in a variety of diseases. This review aims at summarizing and discussing the role of IL‐37 in cardiovascular diseases.

**Methods:**

Data for this review were identified by searches of MEDLINE, Embase, and PubMed using appropriate search terms.

**Results:**

IL‐37 is a newly identified cytokine belonging to the IL‐1 family and is expressed in inflammatory immune cells and several parenchymal cells. It has potent anti‐inflammatory and immunosuppressive properties, with two mechanisms underlying this function. IL‐37 is produced as a precursor and then cleaved into mature form in the cytoplasm by caspase‐1, translocating to nucleus and suppressing the transcription of several pro‐inflammatory genes by binding SMAD‐3. Besides, IL‐37 can be secreted extracellularly, and binds to IL‐18Ra chain and recruits Toll/IL‐1R (TIR)‐8 for transducing anti‐inflammatory signaling. IL‐37 is upregulated in an inducible manner and negatively regulates signaling mediated by TLR agonists and pro‐inflammatory cytokines. The cytokine has been shown to inhibit both innate and adaptive immunological responses, exert antitumor effects, and act as a prognostic marker in a variety of autoimmune diseases.

**Conclusions:**

Recent studies have suggested that IL‐37 plays a role in cardiovascular diseases. In this review, we provide an overview of the cytokine biology, discuss recent advances made in unraveling its cardio‐protective effects, and suggest guidelines for future research.

## Introduction

Interleukin‐37 (IL‐37) was originally discovered in silico in 2000 by three independent groups 1.It was formerly called as IL‐1 family member 7 (IL‐1F7), or referred to as FIL‐1ζ/IL‐1H4/IL‐1H/IL‐1RP1, until more recently it was named as IL‐37 2, 3. IL‐1F members can be categorized into three subfamilies: IL‐1, IL‐18, and IL‐33. IL‐37 belongs to the IL‐18 subfamily, which only contains IL‐18 and IL‐37 1, 4.

As a newly identified member of the IL‐1 family, IL‐37 consists of 12 β‐barrel strands that has IL‐18‐like structural pattern 1. The cytokine is expressed at low levels in various tissues in the body, including tonsils, esophagus, placenta, melanoma, breast, brain, colon, prostate, as well as heart, among others 1, 4. Many cells, including epithelial cells, keratinocytes, renal tubular epithelial cells, monocytes, activated B cells, plasma cells, DCs, macrophages, and CD4+ Tregs, are found to express IL‐37 1, 4, 5, 6. The cytokine can be upregulated in an inducible manner by IL‐1FR ligands and TLR agonists, while IL‐4, IL‐12, IL‐32, and GM‐CSF downregulate its expression 7.

There are five transcripts for the human IL37 gene (IL‐37a‐e), among which IL‐37b is the largest cytokine member and is encoded by five of the six exons spanning the gene 8. IL‐37a and d may be functional, which is undetermined. IL‐37 c and e are non‐functional due to their abnormal folding 9. It is reported that mature IL‐37b can translocate into the nucleus via a caspase‐1‐dependent process 10. Consequently, IL‐37b is the isoform that is biologically functional and can produce homodimers. The protein processing starts with the production of initial precursors, whose signal sequence lacks the pro‐peptide domain 11. The IL‐37 precursor has been observed to be abundant in the cytoplasm. It depends on caspase‐1 to cleave the peptide to turn into mature form 12. Both the precursor and the mature cleaved forms of IL‐37 are biologically active; however, the mature form binds the receptor more efficiently than the precursor form does 13.

The primary function of IL‐37 is to reduce excessive inflammatory responses in a negative feedback mechanism that plays an important role in both innate and adaptive immune systems 14. There are two mechanisms for this effect. The first mechanism is based on SMAD‐3, a member of the SMAD family of transcription factors/regulators that play a role in transduction of TGF‐β1‐mediated signals. Importantly, about 25% of the cleaved/mature form of IL‐37 binds with SMAD‐3 in the cytoplasm, translocates to the nucleus, and inhibits transcription of genes for several pro‐inflammatory cytokines and chemokines, such as TNF‐α, IL‐6, among others 3. The second mechanism is based on IL‐37–IL‐1R8–IL‐18Rα complex. It is demonstrated that after binding with the IL‐18Ra chain, IL‐37 recruits TIR‐8/IL‐1R8/SIGIRR, assembles on the cell surfaces, and forms the tripartite complex. There may be other accessory proteins such as TIGIRR‐ 1 and TIGIRR‐2 that may also be recruited, which leads to the activation of the anti‐inflammatory cascade 15. The tripartite complex increases the activity of STAT3 and PTEN and inhibits transcription factor NF‐kB. The enhancement of STAT3 activity induces the polarization of macrophages and DCs from a pro‐inflammatory state toward an anti‐inflammatory state. Simultaneously, PTEN inhibits the PI3K/Akt/mTOR pathway, thus inhibiting NF‐kB and pro‐inflammatory cytokine production, including IL‐6, TNF‐α, and IL‐1β. IL‐37–IL‐1R8–IL‐18Rα complex also inhibits the NF‐kB pathway by inhibiting an adaptor kinase‐ TAK1 15, 16.

Many papers have reported upregulation of IL‐37 in human diseases, including acute coronary syndrome 17, rheumatoid arthritis 18, 19, and hepatocellular carcinoma 20. In addition, downregulation of IL‐37 is also associated with diverse diseases including psoriasis 21 and asthma 22. From the researches done so far, the anti‐inflammatory characteristics of IL‐37 have made it a potential therapeutic target for autoimmune diseases, acute or chronic inflammatory disorders, and cancer.

## The Role in Cardiovascular Diseases

There have been a multitude of studies done to look into the role of IL‐37 in cardiovascular diseases (reviewed in Table 1).

**Table 1:** The studies and mechanism of IL‐37 in cardiovascular diseases

Disease model	Effect	Reference
Atherosclerosis	↑M1→M2 ↓ NF‐κB, ↓ICAM‐1↓Inflammatory cytokines↓Calcification areas	26, 28, 38, 39
Myocardial infarction	↑Tregs, ↓MPO, ↓ROS↓Myocardial fibrosis↓Cardiomyocyte apoptosis↓Infarct size↑Left ventricular function	17, 44, 46
Ischemia/reperfusion (I/R)	↓Inflammation↑Tregs ↑FoxP3, and CTLA‐4↓Cardiomyocyte apoptosis↓Infarct size↑Left ventricular function	60, 61

An increase in IL‐37 expression has been seen in certain cell types involved with cardiovascular diseases 4, 23, which has made IL‐37 a potential target in pathogenesis. This present review discusses the molecular mechanisms encompassing experimental models and human beings and provides the first comprehensive summary of how IL‐37 plays a protective role in cardiovascular diseases.

### Atherosclerosis

Atherosclerosis is considered a chronic inflammatory disorder in many ways. It is characterized by extensive accumulation of cells, cholesterol, and extracellular matrix, which result in the formation of atherosclerotic plaque in the intima and hardening of the arterial wall 24. It is demonstrated that IL‐37 ameliorated inflammatory responses in epithelial cells, macrophages, and dendritic cells, indicating its potential role in atherosclerosis 23. Endothelial dysfunction caused by inflammation is a key initiating event in atherosclerotic plaque formation 25. Xie et al. 26 found IL‐37 decreased both NF‐κB and ICAM‐1 expression upon TLR2 activation in human coronary artery endothelial cells (HCAECs). The suppression may be due to the inhibitive effect of IL‐37 on NF‐κB 3. This result suggests IL‐37 could prevent atherosclerosis by inhibiting inflammation in endothelial cells. Activated macrophages accumulate in atherosclerotic lesions and play an indispensable role throughout the different stages of atherosclerosis, from the occurrence of fatty streaks to plaque rupture and thrombosis 27. M1/M2 cell differentiation plays a critical role in the pathogenesis and progression of atherosclerosis 23. Boraschi et al. 11 found that IL‐37 was expressed in the foam‐like cells of atherosclerotic coronary and carotid artery plaques, suggesting that IL‐37 is associated with the activation of macrophages and the shift from macrophages to foam cells. Huang et al. 28 found IL‐37 effectively decreased the area ratio between the aorta plaque and vascular cavity. They also observed IL‐37 inhibited M1 macrophages induction from peripheral monocytes by ox‐LDL and facilitated the transformation of macrophages into M2 cells 28. These results indicate that IL‐37 may prevent atherosclerosis by modulating macrophage polarity. In addition, the activation of mature DC promotes the secretion of pro‐inflammatory cytokines and is critical for T‐cell activation and the production of Th1 and Th17 cytokines, which possesses potentially pathogenic properties in atherosclerosis and atherosclerosis related disease, whereas immature DC have been found to secrete anti‐inflammatory cytokine IL‐10, induce the generation of regulatory T cells and therefore effectively ameliorate atherosclerosis 29. IL‐37 can modulate the maturation of DC 30, suggesting its protective effect on atherosclerosis via influencing DC.

Blocking the effects of IL‐18 reduces the atherosclerotic lesion size and induces a switch to a stable plaque phenotype, whereas both endogenous and exogenous IL‐18 accelerated atherosclerosis development 31, 32, suggesting IL‐37 may play a protective role in atherosclerosis via inhibiting IL‐18. Animal experiments have confirmed that pro‐inflammatory cytokines, such as tumor necrosis factor (TNF)‐α and IL‐6, promote the differentiation of vascular smooth muscle cells (VSMCs) into osteoblast‐like cells and exacerbate the arterial calcification process 33, 34. Clinical studies have found that circulating inflammatory mediators, such as C‐reactive protein (CRP) and TNF‐α, are independently associated with an increased incidence of arterial calcification 35, 36. A recent growing body of evidence indicates that RANKL‐induced arterial calcification is mediated by IL‐6 and TNF‐α 37. Higher concentrations of IL‐37 were detected in calcified samples, compared with that in normal arteries, and macrophages and vascular smooth muscle cells were the main source of IL‐37 38. Animal experiments done by Chai et al. 39 also suggested IL‐37 significantly limited calcification areas and decreased plaque size of the atherosclerotic lesions. These results indicated that IL‐37 could attenuate not only atherosclerosis, but also vascular calcification. Therefore, IL‐37 may play a protective role in atherosclerosis through inhibition of inflammatory cytokines production and suppression of macrophage and DC activation 23.

### Myocardial infarction

Recent study revealed that IL‐37 level increased obviously in peripheral blood of acute myocardial infarction (MI) patients 17. Acute MI can cause serious myocardial ischemia, thus activating autoimmunity, which recruits a large number of inflammatory cells to the infarction area and releases plenty of cytokines participating in inflammatory response 40. Excessive inflammatory cytokines are demonstrated to produce toxic effect on myocardial cells, accelerate myocardial cell apoptosis and, eventually impair the heart function 41. Numerous inflammatory cells infiltration may also release abundant inflammatory mediators such as MPO to participate in inflammation. These inflammatory mediators take part in myocardial cells necrosis and apoptosis, as well as endothelial cell dysfunction 42. It was also reported that MPO enzyme was an independent predictor for acute coronary artery syndrome, closely associated with acute MI 43. Studied done by Xu et al. 44 indicated that IL‐37 played an anti‐inflammatory role by inhibiting MPO expression in acute MI mice.

Excessive immune‐mediated inflammatory reactions can cause myocardial cell hypertrophy and apoptosis, and affect the myocardial systolic function, therefore leading to the occurrence of ventricular remodeling and heart failure 45. It is indicated by Zhu et al. 46 that IL‐37 decreased infarct size and myocardial fibrosis and inhibited cardiomyocyte apoptosis. This effect is possibly via the increased Tregs induced by tolerogenic DCs 46.

It also proved the activation of NF‐κB signaling pathway after MI, and the inhibition of this signaling pathway can improve cardiac function after MI and prognosis 47. NF‐κB is an important nuclear transcription factor, as it not only plays an important role in inflammation, but also relates to myocardial cell apoptosis and myocardial remodeling process after MI 48. Given the fact that IL‐37 could modulate the expression of NF‐kB, IL‐37 may attenuate remodeling after acute MI through the inhibition of NF‐kB.

Angiogenesis, the formation of new blood vessels from pre‐existing vasculature, is tightly controlled by pro‐angiogenic and anti‐angiogenic cytokine 49. Angiogenesis was promoted by many cytokines of the interleukin‐1 (IL‐1) family, such as IL‐1α, IL‐1β, IL‐18, and IL‐33 50, 51, 52. Different cytokines seem to have a role in angiogenesis through the use of different pathways to regulate the immune microenvironment 53. Yang et al. 54 found that the upregulation of IL‐37 under hypoxic conditions enhanced endothelial cell proliferation, capillary formation, migration, and vessel sprouting from aortic rings. Further showed, in the mouse model of retinal vascular development, neonatal mice administrated with IL‐37 displayed increased neovascularization 54. These findings are similar to those from the studies done by Zhao et al. 55, the only contradiction is that Zhao et al. found the IL‐37 pro‐angiogenic effect correlated with level of VEGF‐A and Ang‐2, but the studies done by Yang et al. 54 showed the expression of IL‐37 in HUVECs was not affected by VEGF. The discrepancy may be due to the different types of cells and the different concentrations of treated IL‐37. In addition, it was also found that serum IL‐37 level had a negative correlation to VEGF and Ang‐2 levels. The tube formation of HUVECs was suppressed by the rhIL‐37 pretreatment 56. The biphasic pro‐angiogenic effect of IL‐37 might be due to its different expression level. In summary, IL‐37 protects MI and contributes to heart function by reducing infarction size, as well as to the prognosis of remolding by inhibiting NF‐kB and promoting angiogenesis.

### Ischemia/reperfusion (I/R) injury

IR injury is caused by hypoxia and cessation in blood flow, followed by an intense inflammatory response upon reperfusion 57. Pro‐inflammatory chemokines and cytokines, like TNF‐α, macrophage inflammatory protein (MIP‐2) and KC, are involved in reperfusion injury 58. A study was done to investigate the effects of IL‐37 on hepatocytes and hepatic inflammation induced by ischemia/reperfusion (I/R) 59.This study found that the production of these pro‐inflammatory mediators was reduced in vivo with IL‐37 treatment, and the successive neutrophil recruitment was also weakened. Our study also found that mice treated with recombinant human IL‐37 before reperfusion showed I/R injury amelioration, compared with vehicle‐treated mice 60.The size of the infarcted area was decreased, cardiac troponin T levels were reduced, and cardiac function was improved in the IL‐37 treated mice. The protective properties of IL‐37 against I/R injury were attributed to the suppression of pro‐inflammatory cytokine, chemokine, and neutrophil infiltration, which resulted in a reduction of ROS production and cardiomyocyte apoptosis. In addition, it was also found that TLR‐4 expression and NF‐κB activation were inhibited by IL‐37 after I/R while IL‐10 level was increased .

In keeping with that, the study carried out by Xu et al. showed that IL‐37 had protective effect on myocardial infarction microcirculation reperfusion injury, the possible mechanism may be to promote Treg cells, inhibit inflammatory reaction (decreased IL‐6 and TGF‐α) and the expression of CTLA‐4 and FoxP3 61. On the basis of this ability to modulate a number of cytokines, IL‐37 may be a novel therapeutic candidate for myocardial I/R injury.

## Conclusion

Although IL‐37 is a novel interleukin in the field of immunology, it has been found to be a key regulator in both innate and adaptive immunities. Even with all the IL‐37 researches done since its discovery in cardiovascular diseases, much still needs to be elucidated. The mechanism of action by which it exhibits its anti‐inflammatory and cardio‐protective properties has yet to be completely determined. In addition to its anti‐inflammatory and immune‐deviatory effects, IL‐37 also exerts effects on metabolic activity both on the cellular and organismal levels. IL‐37 transgenic mice are resistant to the metabolic effects caused by LPS. The transgenic mice are also relatively less susceptible to obesity‐induced inflammation and insulin resistance 62. These results suggest the potential role of IL‐37 in diabetic cardiomyopathy. Whether IL‐37 exerts a protective role in hypertension, autoimmune myocarditis, and heart failure has not been clearly investigated. Therefore, further studies are needed to fully understand the therapeutic potential of IL‐37.

## Conflict of Interest

None declared.

## Ethical Statement

No humans or animals were involved in the study—no ethical approval was required for this manuscript.
